# Plasma Membrane Lipid Domains as Platforms for Vesicle Biogenesis and Shedding?

**DOI:** 10.3390/biom8030094

**Published:** 2018-09-14

**Authors:** Hélène Pollet, Louise Conrard, Anne-Sophie Cloos, Donatienne Tyteca

**Affiliations:** CELL Unit, de Duve Institute & Université Catholique de Louvain, UCL B1.75.05, Avenue Hippocrate, 75, B-1200 Brussels, Belgium; helene.pollet@uclouvain.be (H.P.); louise.conrard@uclouvain.be (L.C.); anne-sophie.cloos@student.uclouvain.be (A.-S.C.)

**Keywords:** microvesicle, cytoskeleton, cholesterol, ceramide, sphingomyelinase, raft, lipid domains, calcium, oxidative stress, red blood cell

## Abstract

Extracellular vesicles (EVs) contribute to several pathophysiological processes and appear as emerging targets for disease diagnosis and therapy. However, successful translation from bench to bedside requires deeper understanding of EVs, in particular their diversity, composition, biogenesis and shedding mechanisms. In this review, we focus on plasma membrane-derived microvesicles (MVs), far less appreciated than exosomes. We integrate documented mechanisms involved in MV biogenesis and shedding, focusing on the red blood cell as a model. We then provide a perspective for the relevance of plasma membrane lipid composition and biophysical properties in microvesiculation on red blood cells but also platelets, immune and nervous cells as well as tumor cells. Although only a few data are available in this respect, most of them appear to converge to the idea that modulation of plasma membrane lipid content, transversal asymmetry and lateral heterogeneity in lipid domains may play a significant role in the vesiculation process. We suggest that lipid domains may represent platforms for inclusion/exclusion of membrane lipids and proteins into MVs and that MVs could originate from distinct domains during physiological processes and disease evolution.

## 1. Introduction

**Figure 1:**
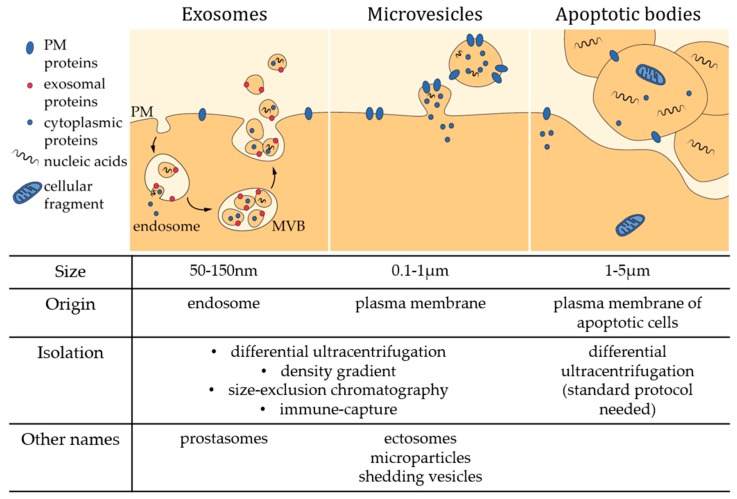
Characteristics of the three main classes of extracellular vesicles. MVB, multivesicular body; PM, plasma membrane.

In recent decades, the field of transcellular signaling has been revolutionized by the emerging concept of signal transmission through extracellular vesicles (EVs). For a long time, vesicles seen in intercellular spaces by electron microscopy were thought to be artifacts or inert cellular fragments resulting from damaged cells in the vicinity. Nonetheless, all cells, from bacteria to plants and animal cells, seem to have the ability to produce EVs [1,2]. However, there is still no real consensus regarding EV classification and nomenclature [3], probably due to the variety of EV size, composition, origin and targets, but also due to difficulties related to their isolation and analysis (see Section 2). Most reviews classify EVs into three groups: exosomes, microvesicles (MVs) and apoptotic bodies (Figure 1) [4,5,6,7]. Exosomes are the smallest EVs (50–150 nm in diameter) and are released upon multivesicular bodies exocytosis. MVs are produced by direct local deformation and budding of the plasma membrane (PM) leading to vesicles of more heterogeneous and bigger size (100 nm–1 µm in diameter). It should be noted that these vesicles are often given other names, including ectosomes, microparticles, shedding vesicles or oncosomes (in the particular case of cancer cells). Apoptotic bodies (1–5 µm in diameter) are generated by blebbing of cells undergoing apoptosis. However, this classification should be taken with caution as most of the currently used techniques only make it possible to separate small EVs enriched in exosomes from large EVs enriched in MVs (see Section 2) [8]. Yet, other studies have evidenced by imaging the budding from the PM of vesicles with size closer to that of exosomes [9,10,11]. In this review, we will focus on PM-derived vesicles whatever their size.

Extracellular vesicles have been shown to contribute to a large variety of pathophysiological processes including red blood cell (RBC) senescence [12], coagulation [13], inflammation [13,14], migration [15], tumorigenesis [16] and infection [17]. As they are found in body fluids (e.g., blood, urine, cerebrospinal fluid, milk), they are easily accessible and might represent useful diagnostic biomarkers and/or targets for therapeutic applications (reviewed in [18]). In this review, we will focus on vesicles derived from the PM of RBCs, platelets, immune cells, nervous cells and tumor cells. Before providing detailed information regarding their biogenesis, we present below a short non-exhaustive overview of their pathophysiological effects.

**Figure 2:**
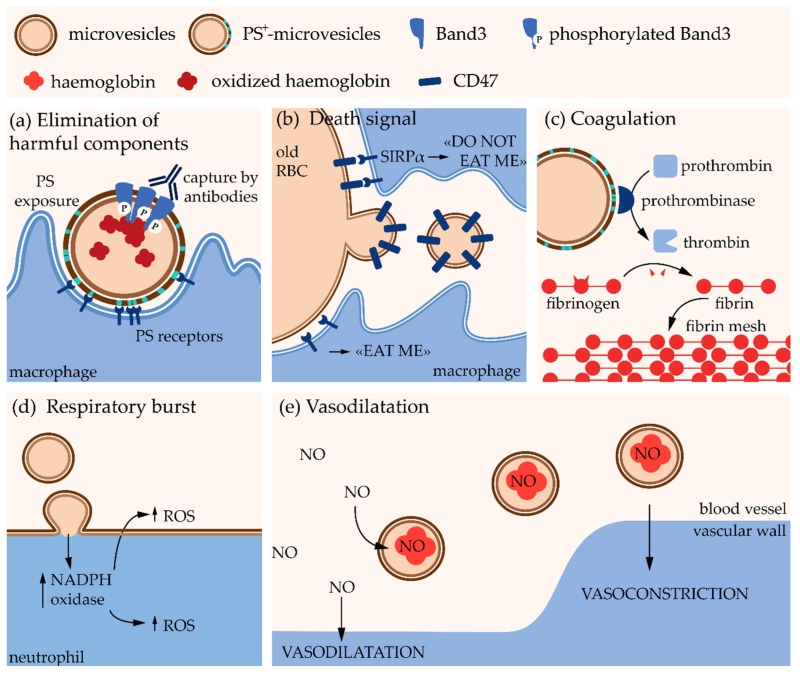
Pathophysiological effects of erythrocyte-derived microvesicles. PS, phosphatidylserine; RBC, red blood cell; SIRPα, signal regulatory protein α; NADPH; reduced form of nicotinamide adenine dinucleotide phosphate; ROS, reactive oxygen species; NO, nitric oxide.

Erythrocytes undergo multiple changes during their 120-day lifespan in the circulation, including the decreased activity of multiple enzymes, the gradual accumulation of oxidative damage, the redistribution of ions, the loss of membrane by vesiculation as well as cell volume, density and deformability alterations (for reviews, see [19,20,21]). Microvesicle generation constitutes a central mechanism in the RBC homeostasis and is responsible for the loss of ~20% of the PM while the hemoglobin concentration increases by ~14% [22,23]. Microvesicles have been proposed to contribute to RBC senescence by two opposite mechanisms. On one hand, they protect RBCs from premature elimination via transport of molecules that could induce recognition by the reticuloendothelial system such as non-functional hemoglobin, oxidized and aggregated Band3 and oxidized proteins [23] (Figure 2a). On the other hand, they appear to contain CD47, a self-marker that prevents the recognition and clearance of RBCs by macrophages. Elimination of CD47 from the RBC membrane through selective shedding could then promote the removal of old RBCs [24] (Figure 2b). Studies on mice suggest that MVs from RBCs are removed very fast from the circulation by the reticuloendothelial system [25] because they can have deleterious effects on other cells. For instance, MVs bear at their external leaflet phosphatidylserine (PS), which acts as an “eat me” signal for macrophages but also promotes coagulation, as PS enhances prothrombinase activity and other coagulation factors (Figure 2a,c). Moreover, RBC-derived MVs induce an excessive production of reactive oxygen species (ROS) in neutrophils and could be responsible for exhortation of the respiratory burst, i.e., the rapid release of ROS necessary to answer to an infection [26] (Figure 2d). Finally, they contain hemoglobin, which allows them to bind nitric oxide modifying thereby bioavailability of the latter for vascular homeostasis regulation [27] (Figure 2e). However, these effects have been shown with MVs isolated from blood storage. Although the biological content should reflect the functional properties of circulating MVs, further investigations in vivo are needed to confirm these hypotheses. Nevertheless, the properties described above could partly explain reduced post-transfusion efficacy and increased risk of adverse reactions in patients after transfusion [28,29].

Platelet is another blood cell type able to release a high quantity of EVs. Platelet EVs represent ~30% of the blood EVs [30] and, in contrast to RBCs, these include MVs as well as exosomes. Platelet-derived vesicles are essential for the regulation of the hemostasis, as revealed by the bleeding disorders caused by the decreased formation of platelet-derived MVs in patients with Scott Syndrome [31]. Their pro-coagulant activity is due to the exposition on their surface of pro-coagulant molecules, such as PS and tissue factor (TF), one initiator of the coagulation cascade [30]. Additionally, platelet-derived MVs promote cell proliferation, survival and migration, which are essential for endothelial repair and wound healing [32]. They are also effectors of the immune response by increasing monocyte adhesion, promoting inflammatory pathways and cytokine release in monocytes and endothelial cells (ECs) [33], stimulating antigen-specific IgG production [34] and upregulating neutrophil aggregation, activation and phagocytic activity [35].

Monocytes and neutrophils themselves are also able to release MVs and exosomes, producing autocrine effects or serving as mediators to communicate with other cells (reviewed in [36,37]). They can have either pro-inflammatory or anti-inflammatory effects [38]. They also promote blood coagulation through exposure of PS and TF in monocyte MVs and through direct interaction between neutrophil MVs and platelets, which promotes platelet activation [39] and TF expression [36].

In the central nervous system, only exosome-like EVs have been described in neurons, oligodendrocytes and Schwann cells in physiological conditions [40]. On the other side, microglia cells (i.e., the macrophage resident cells) and astrocytes (i.e., support cells, also involved in the blood brain barrier) have been shown to release both exosomes and MV-like EVs [41]. The major MVs described are released by microglia cells and astrocytes upon ATP activation of P_2_X_7_ receptors and contain the interleukin-1β which is released at the site of tissue damage to initiate an acute inflammatory response [42]. Extracellular vesicles also appear to play critical role in neurodegenerative diseases. For example, in Alzheimer disease, MVs released by microglia promote the amyloid-β pathogenesis by increasing the solubility of the misfolded protein (i.e., the soluble form is more neurotoxic than the aggregated one) [43]. Moreover, as microglia-derived MVs regulate the inflammatory response, they have been shown to be increased and to play major role in multiple sclerosis, a form of neuroinflammation [44]. Those MVs carry inflammation factors that promote the degradation of the extracellular matrix and tight and adherens junctions, leading to the disruption of the blood brain barrier [45].

Cancer cell-associated vesicles were reported for the first time in 1978 in patients suffering from Hodgkin disease. Since then, evidence has been accumulating that tumor-derived MVs constitute important players in cancer initiation and progression through communications between cancer cells. Microvesicles also facilitate intercellular communication between cancer cells and microenvironmental cells (e.g., stromal, immune and vascular cells), either located directly in the primary tumor-environment or at distance, promoting pre-metastatic niche formation. Microvesicles are implicated in several stages of tumorigenesis and metastasis by increasing angiogenesis and extracellular matrix remodeling, promoting escape from the immune system, inducing resistance to therapy and triggering blood coagulation (reviewed in [16,18,46,47]). Because of the multiple roles of MVs in cancer, they could be seen as prognostic and/or diagnostic biomarkers. Hence, they could represent emerging targets for cancer therapy (reviewed in [18]).

## 2. Microvesicle Isolation and Characterization

Several methodologies have been developed over the years to optimize EV isolation. Differential ultracentrifugation is the most frequent one, even if protocols can considerably vary in terms of speed and time intervals. Increasing centrifugal forces allow to separate EVs from cell debris and intact cells thanks to their difference in size and density [48,49,50,51]. This method makes it possible to reach a recovery of up to 80% and offers the possibility to process large volumes without the need for chemicals that could interfere with downstream analysis [50]. Among limitations, one can cite EV aggregation, contamination by protein aggregates and viruses as well as EV damaging during high-speed centrifugation [48]. Moreover, since it is moderately time-consuming and the equipment is expensive, it is not considered to be a clinically applicable isolation technique. Another method based on centrifugation is the density gradient centrifugation, which presents a lower recovery of 10–50% but avoids protein contamination of samples thanks to density differences [48,52]. Nevertheless, co-isolation of lipoproteins cannot be excluded. For the same reasons as for differential ultracentrifugation, this methodology finds no clinical application [48]. Filtration represents an alternative method that can be applied alone or in combination with ultracentrifugation. However mechanical damage due to the pressure applied for passing EVs through the filter can affect their properties [50]. According to the size cut-off of the column, size-exclusion chromatography allows EV separation from non-aggregated proteins and high density lipoproteins (HDL) but contamination with material of similar size, such as aggregated proteins and viruses, cannot be excluded. The excellent recovery rate (up to 90%), the absence of EV damaging, the low cost and the quickness make this methodology interesting for clinical applications. However, this method is not suitable for large volumes and requires a pre-concentration step by ultracentrifugation [48]. Finally, the affinity-based methods (better known as immune-capturing methods) are based on the interaction of EV surface molecules with antibodies, lectins or lipid-binding proteins, either biotinylated or coupled to magnetic beads. These techniques are fast and simple and contamination of purified sample is minimal. However, it is cost-effective and not adapted to processing large-volume samples [50]. In the face of the large diversity of isolation methods, the quantity and the quality of the starting material must guide the researcher.

Another criterion to consider is the type of analysis downstream of EV purification, including their structural characterization. Here also several techniques exist. Electron microscopy makes it possible to assess EV size and morphology and to identify their cellular origin [48,53]. Since dehydration and fixation required for traditional electron microscopy could possibly lead to EV morphology changes, cryo-electron microscopy is widely recommended. Atomic force microscopy (AFM) also makes it possible to determine EV structural properties via the interaction of a probing tip (cantilever) with the surface of the sample, which generates a 3D-image of the surface topography. However, as for electron microscopy, changes in EV morphology can occur due to the necessity of immobilizing the material [53]. Additionally, one can cite the dynamic light scattering and nanoparticle tracking, which are both based on the same principle. Thanks to the recording of light scattering over time and its modification due to EV Brownian motion, it is possible to determine their size and size distribution. This approach is more reliable when the sample size is homogenous and not polydispersed [53]. For larger EVs, flow cytometry is an alternative technique combining light scattering and fluorescence. The labeling of EVs with fluorescent antibodies/probes allows the specific recognition of surface markers [48,53]. In addition to structural characterization, it is also possible to biochemically characterize the EVs. This is beyond the scope of the manuscript and we invite the reader to refer to the review by Ramirez et al. for more insights regarding techniques for biochemical and in vivo characterization [51].

## 3. Microvesicle Molecular Properties

The abundance and properties of MVs appear to fluctuate depending on the cell origin, the pathophysiological context and also the subject tested (e.g., age, gender, fasting state, medication exposure, physical activities, pregnancy and diseases) [54,55,56,57,58]. Moreover, variations in isolation techniques, culture conditions and methods to stimulate the shedding (e.g., calcium [Ca^2+^] ionophores, lipopolysaccharide, hypoxia, tumor necrosis factor TNFα, ATP) could lead to conflicting data [59]. Finally, most studies related to MV content are based on “MV pellets” obtained by differential ultracentrifugation, which most of the time contain mixed populations (especially when extracted from body fluids) [8]. It is then difficult to provide a digest of the MV content and even more to erect rigid rules. Nevertheless, efforts have been made to collect datasets from many EV studies and put them online (Vesiclepedia; EVpedia) [60,61] and to carefully characterize co-isolated mixed EV populations from “traditional” isolation procedures to refine and determine new optimized protocols [8].

Microvesicles are limited by a lipid bilayer (Section 3.3) and can carry a diversity of proteins (Section 3.1) and nucleic acids (Section 3.2). Although they are expected to exhibit a similar content as the PM from which they derive, accumulated evidence highlights that the MV composition is the outcome of a regulated sorting mechanism at the PM, leading to enrichment or despoliation of some chosen components.

### 3.1. Protein Content

Proteins associated with MV biogenesis are generally found in these vesicles. For instance, the MVs produced by the tumoral LOX cell line are positive for the small GTPase ARF6 known to regulate their release [62]. Likewise, Rab GTPases suspected to play a role in MVs released by neuroblastoma cells associate with these vesicles [63]. Regarding cytoskeleton proteins, actin has been detected in neutrophil- [64] and RBC-derived MVs while spectrin, the structural basis of RBC cytoskeleton, is lacking [65,66]. Proteins known to localize in lipids rafts appear also enriched in some MVs [39,66,67], but it is not a common rule (see Section 5.3). Some MVs, in particular those released by ECs, neutrophils and tumor cells, are charged with proteolytic enzymes, allowing tissue microenvironment remodeling which is essential for angiogenesis, tissue repair or cancer cell invasion. For instance, matrix metalloproteinases are found in large oncosomes from prostate cancer tissues and cells [68,69] and in MVs from human breast carcinoma cells, neutrophils [64] and ECs [70]. For a complete review on metalloproteinases in EVs, see [71]. Adhesion molecules are also commonly found in MVs as they can mediate direct stimulation of the recipient cells or initiate MV internalization. Thus, different classes of integrins are associated with MVs from monocytes/macrophages, neutrophils, platelets, endothelial progenitor cells and tumoral cells [39,72,73,74,75,76]. For example, P-selectin is found in platelet-derived MVs [73] and the P-selectin glycoprotein ligand-1 (PSGL-1) is detected in monocyte/macrophage-derived MVs able to fusion with platelets [39]. Finally, MVs could contribute to the propagation of oncoproteins among the tumoral cells, as it was shown for the oncogenic form of the epidermal growth factor receptor (EGFRvIII) present in aggressive human brain tumors [77].

### 3.2. Nucleic Acid Content

The study of RNA in EV samples represents a growing field of research thanks to technical advances in the detection of scarce and complex RNA samples. Using high-throughput RNA sequencing, various mRNAs and many types of non-coding RNAs have been found in EVs [78]. For instance, MVs from endothelial progenitor cells are loaded with mRNAs associated with the PI3K/Akt signaling pathway, which triggers angiogenesis in ECs and promotes cell survival, proliferation and organization in capillary-like structures [76]. Another example is the transfer of mRNA for growth factors from tumoral-derived MVs to monocytes. This enhances monocyte survival in vitro [79]. Non-coding RNAs might also be present in MVs [80]. For instance, miRNA has been detected in embryonic stem cell MVs [81] and human adult liver stem cell-derived MVs [82]. The presence of far more types of non-coding RNAs has been assessed in mixed EV populations or non-defined EVs [78,80]. However, several investigations have pointed out that, when comparing MVs with exosomes, the latter is the richest reservoir for almost all RNAs [83,84]. Even if that does not mean the RNA transfer through MVs is inefficient, it suggests that transcellular transfer of genetic material is less important in MVs than in exosomes. To the best of our knowledge there is no indication that DNA is present in MVs, but it was already found in apoptotic bodies, exosomes, “exosome-like” vesicles (i.e., unknown origin) and mixed EV populations [85]. However, it is possible to artificially load MVs with plasmid DNA with effective transfer to the recipient [86].

### 3.3. Lipid Content

Lipids are the basic structural constituents of EVs but are the least-studied components and the least-appreciated topic in dedicated reviews. Although EVs have for a long time been mainly distinguished based on their size, origin and protein content, protein-to-lipid ratio has recently been proposed as an alternative criterion, at least until selective markers become available. For instance, apoptotic bodies exhibit the highest protein-to-lipid ratio, followed by MVs and then by exosomes, as revealed by a comprehensive analysis of EV preparations from various myeloid and lymphoid cells lines as well as blood plasma [87]. As lipids present a density of approximately ≈1 g cm^−3^ and proteins of >1.3 g cm^−3^, density gradients (see above Section 2) could therefore be used to separate subpopulations of EVs with differential protein-to-lipid ratios [52].

Focusing on MV lipid content, only a few reliable data can be found in the literature, with sometimes conflicting information (Table 1). Although ceramide (Cer) and sphingomyelin (SM) are enriched in the MVs originating from some tumor cells (namely U87 glioblastoma and Huh7 hepatocellular carcinoma cells) and from human bone marrow-derived mesenchymal stem cells [88], these two sphingolipids (SLs) are less enriched in MVs from platelets and not at all in MVs isolated from plasma or RBCs upon storage. Regarding phospholipid (PLP) content, while PS and phosphatidylethanolamine (PE) appear to be depleted from MVs, phosphatidylcholine (PC) seems to present a similar content as in the PM. No reliable information can be found on cholesterol (chol) content, except in two studies showing no spectacular enrichment. To summarize, some studies reveal that MVs exhibit a similar lipid composition as the PM, while others show specific enrichment/depletion, leading to the suggestion that MVs can shed from specific PM locations. Discrepancies between studies could be related to the cell origin and the pathophysiological context, but also to the MV isolation, purification and characterization conditions.

Remodeling of membrane asymmetry in MVs appears at first glance less debated. Accordingly, PS exposure at the outer leaflet is the most widespread tool to identify EVs. However, it should be noted that Annexin V, a specific tool for outer PM leaflet PS, seems not able to unveil the entire EV population. For instance, by cryo-electron microscopy, Arraud et al. revealed that a large amount of EVs from plasma does not expose PS [30]. Whether this population refers to exosomes remains to be determined. Moreover, Annexin V appears to bind only 20% of unstimulated platelet-derived MVs and its binding on activated platelets depends on the agonist used for platelet activation [96]. Furthermore, severe disruption of protein-protein interactions associated with RBC morphology changes can induce increased MV production without increased PS exposure [97]. Finally, MVs induced upon RBC treatment with sphingomyelinase (SMase) are much more heterogeneous in PS exposure than those generated by spontaneous vesiculation, suggesting distinct mechanisms for biogenesis [98]. Therefore, one should be careful not to discard relevant MV population(s) when using Annexin V as a MV marker. Hence, these observations could suggest that different MV types can be generated by the same cell population depending on its activation state, suggesting distinct mechanisms of biogenesis (see Section 4 and Section 5).

Lipids present in MVs could also act as messengers (for a complete review [99]). For instance, diacylglycerol and PLPs of MVs released from platelets, RBCs, ECs and thymocytes can be hydrolyzed by phospholipases A_2_ to release a polyunsaturated fatty acid that is necessary for eicosanoids (i.e., lipophilic hormones) production [100]. In vivo, phospholipases A_2_ could trigger the incorporation of the target MVs to the recipient cells with the help of the produced eicosanoids. For instance, in rheumatoid arthritis, the concerted action of secreted phospholipase A_2_ enriched in inflamed joint fluid and platelet-type 12-lipoxygenase present in platelet-derived MVs produces an eicosanoid (the 12(S)-hydroxyeicosatetranoic acid) which triggers the fusion of MVs with the neutrophil membrane [101].

## 4. Microvesicle Biogenesis and Shedding—General Mechanisms

In this section, we describe in detail the mechanisms involved in RBC microvesiculation. We then provide some clues on nucleated cells.

### 4.1. Red Blood Cells

#### 4.1.1. Main Determinants of Red Blood Cell Integrity Maintenance

Through its life span, alterations of one or several factors that regulate RBC deformability will rapidly affect the RBC integrity and therefore initiate MV shedding from the membrane. Four major factors regulating the RBC deformability have been described: (i) the cytoskeleton structural properties and its vertical interactions with the membrane; (ii) the cytoplasmic viscosity; (iii) ion balance and subsequent volume regulation; and (iv) metabolic processes controlling ATP levels and redox state.

**Figure 3:**
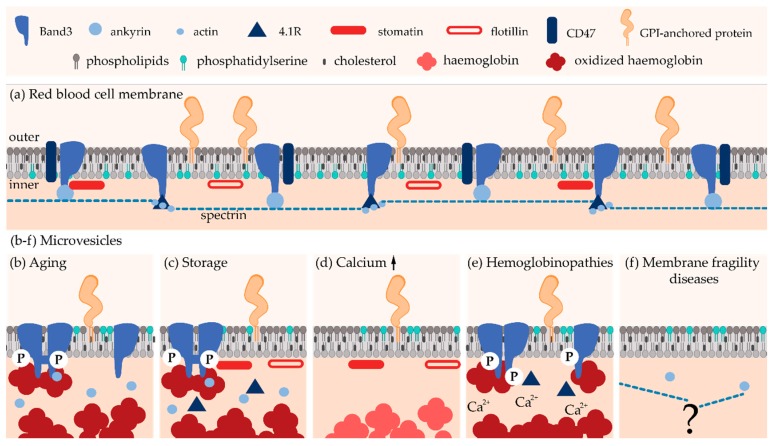
Schematic representation of lipid and protein composition of red blood cell-derived microvesicles. (**a**) RBC plasma membrane. (**b**–**f**) RBC-derived microvesicles in (**b**,**c**) physiological processes (senescence in vivo and storage at 4 °C), (**d**) pharmacological Ca^2+^ boost, and (**e**,**f**) pathological situations (hemoglobinopathies and membrane fragility diseases).

First, the RBC cytoskeleton strengthens the lipid bilayer and endows the membrane with durability and flexibility to survive in the circulation [102]. It is made of a pseudohexagonal meshwork of spectrins linked to the membrane by two multiprotein anchorage complexes: the ankyrin and the 4.1R complexes (Figure 3a). Ankyrin links the spectrin tetramers to the membrane through association with the membrane channel Band3. This complex is completed by association with the “marker of self” CD47, among others. 4.1R forms the second anchorage complex with actin and Band3, *inter alia* (for a complete review see [102]). The modulation of the interactions between cytoskeleton and membrane is tightly regulated by protein phosphorylation [103,104,105], association with PLPs [106,107] and Ca^2+^ [108], among others.

Second, RBC cytoplasmic viscosity, determined by hemoglobin concentration (comprised between 32 and 36 g/dL [109]) and state (i.e., polymerization, crystallization, degradation and oxidation [110]), is finely regulated.

Third, RBC ion balance and subsequent volume control is regulated by ion channels, symporters, antiporters and pumps. Among ion channels, one can cite Piezo1, a mechanosensitive non-selective cation channel recently identified as the link between mechanical forces, Ca^2+^ influx and RBC volume homeostasis. The Ca^2+^-activated K^+^ channel (named Gardos), the Cl^−^/HCO_3_^−^ antiporter Band3 and the plasma membrane Ca^2+^ ATPase pump (PMCA) are also essential for the RBC homeostasis. For additional information regarding the regulation of RBC hydration and volume, please refer to [110,111].

Fourth, deformability of RBCs is affected by metabolic processes controlling ATP content and redox state. Intracellular ATP represents an energy source needed for (i) ion pumps like Na^+^/K^+^- and Ca^2+^-ATPases, ATP-dependent glucose transporters, flippases and floppases; (ii) modulation of the compliance of the membrane with the cytoskeleton; and (iii) de novo synthesis of glutathione that is essential for the antioxidant system [104,112,113,114]. The extensive antioxidant system in RBC is designed to neutralize the harmful ROS generated through the constant exposure to variable oxygen pressures. Indeed, the major source of RBC oxidative stress is hemoglobin redox reactions. The reactive free radical species generated by hemoglobin reactions and the interactions of hemoglobin with membrane and cytoskeleton proteins both induce oxidative stresses and are involved in RBC aging. In addition, exogenous oxidants enter the RBC and react with hemoglobin [115]. The main antioxidant protein is the glutathione which presents two forms: the reduced GSH and oxidized GSSG. GSH scavenges ROS and reacts with another glutathione to form the inoffensive GSSG. The GSH pool is then restored by the action of the glutathione reductase and the reduced form of nicotinamide adenine dinucleotide phosphate (NADPH) [19].

#### 4.1.2. Microvesicles upon Red Blood Cell Senescence, Blood Storage and Intracellular Calcium Boost

In plasma, RBC-derived MVs are a homogeneous population of ~150 nm in diameter [116]. Regarding composition, RBC-derived MVs from the plasma of healthy individuals (i) exhibit a very high content of Band3 and actin, contrasting with a lack of spectrin and ankyrin, (ii) are enriched in enzymes involved in redox homeostasis and in irreversibly modified hemoglobin, (iii) present PS at their outer lipid leaflet, and (iv) contain the glycosylphosphatidylinositol (GPI)-anchored proteins CD55 and CD59 (Figure 3b; reviewed in [21]).

During blood storage, remodeling of the RBC membrane is associated with the oxidative cross-linking and subsequent loss of Band3, lipid raft rearrangement and loss, as well as caspases activation [117]. Accordingly, RBC storage-derived MVs (i) accumulate oxidized and clustered Band3 and actin but lack spectrin, (ii) contain aggregated hemoglobin, (iii) expose PS at the surface, and (iv) contain the GPI-anchored proteins acetylcholinesterase and CD55 as well as stomatin and flotillins [65,66].

As the features of MVs stored in vitro are reminiscent of those of aging-released MVs, one can suggest a similar if not identical mechanism of shedding, even though some aspects of RBC aging in vivo may be more pronounced in blood bank RBC concentrates [118]. However, the loss of Band3 and several raft proteins from the RBC membrane upon storage seems to occur with distinct kinetics [117], suggesting several distinct vesiculation processes during storage. In agreement with this hypothesis, RBC-derived MVs upon storage present size and total protein content that increase over time. Moreover, the oxidation index of the MVs is very high before 3 weeks of storage, then abruptly decreases. Finally, while the vesicles contain apoptosis-related signaling molecules after day 10 of storage, the presence of CD47 is only visible from day 17 [65]. Our unpublished data also suggest multiple vesiculation processes during RBC storage.

Two non-mutually exclusive mechanisms have been proposed in the literature to explain MV release from RBCs:

•  Band3 Model

Accumulation of Band3 and actin, which contrasts with the absence of spectrin in MVs generated upon RBC aging and blood storage, supports the hypothesis that partial membrane:cytoskeleton uncoupling, due to the breakage of ankyrin:Band3 binding, could contribute to the vesiculation process [21]. Accordingly, a simulation study highlighted that a significant reduction of the local anchorage density is required for vesiculation [119]. Furthermore, it has been shown that cytoskeleton stiffness and density both increase upon RBC senescence, leading to larger compressive forces on the cell membrane. These cytoskeleton modifications have been hypothesized to result from the vesicle detachment from the membrane and the subsequent increased membrane curvature [120,121,122].

**Figure 4:**
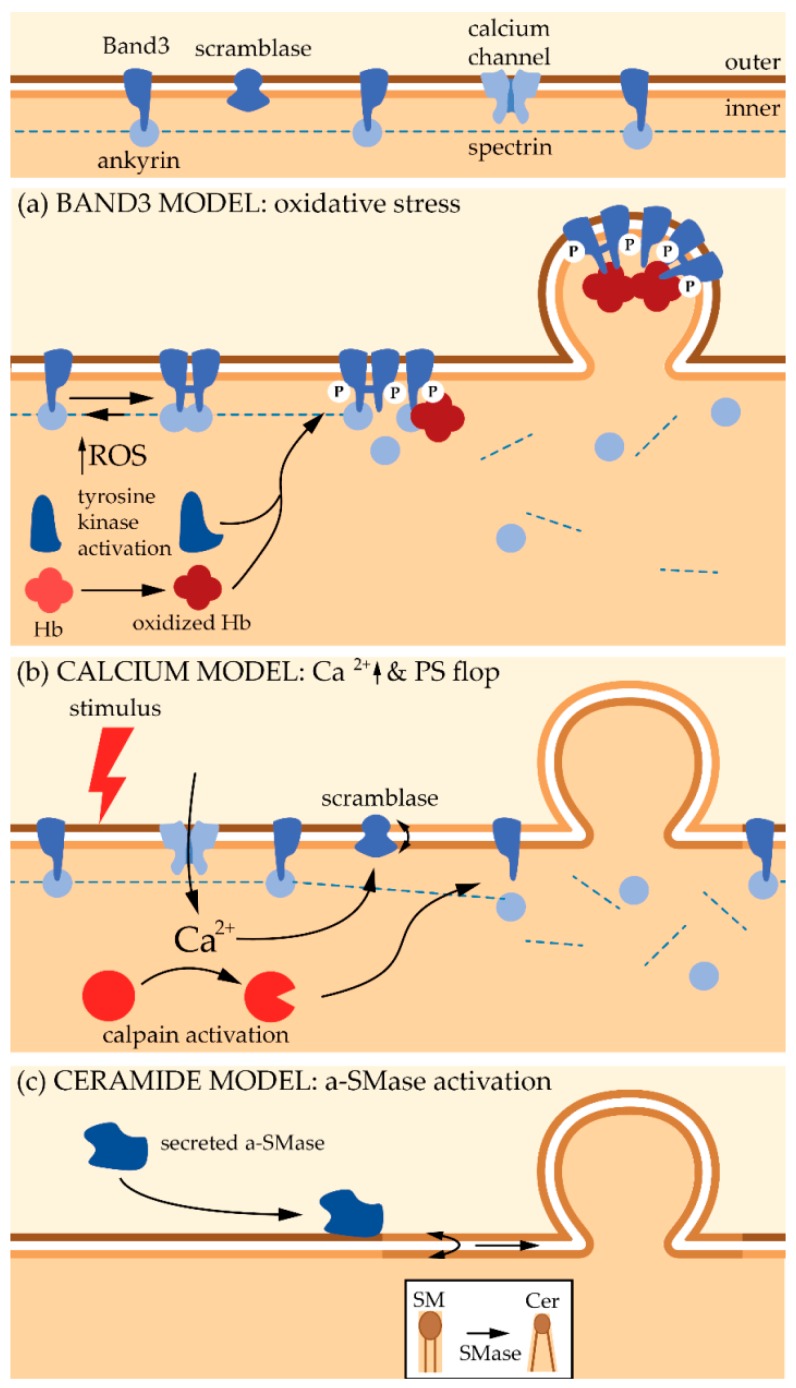
Models described in the literature for the biogenesis and shedding of red blood cell-derived microvesicles. Hb: haemoglobin; P: phosphorylation; a-SMase: acid sphingomyelinase.

However, cytoskeleton instability is probably not the primary event leading to vesiculation. Indeed, MV enrichment in enzymes involved in redox homeostasis and in irreversibly modified hemoglobin suggests that oxidative damage also contribute to the vesiculation process. Bosman et al. even suggested that this constitutes the primary trigger for vesiculation [21]. The increase in oxidative stress during RBC senescence results from a decrease in the anti-oxidative defense due to a lower activity of superoxide dismutase, catalase, glucose-6-phosphate dehydrogenase and aspartate aminotransferase. These latter two enzymes are involved in the formation of anti-oxidant glutathione (GSH) and NADPH molecules [123,124,125]. Oxidative stress appears to lead to the clustering of Band3 thanks to two mechanisms (Figure 4a). First, ROS activate Src tyrosine kinases, which in turn induce phosphorylation of Band3. Accordingly, hyper-phosphorylation of Band3 has been evidenced upon RBC aging and storage [126]. This phosphorylation in turn induces Band3 detachment from membrane skeleton, most probably by disruption from ankyrin, increasing its mobility and its clustering [126]. Second, ROS induce oxidation of hemoglobin into hemichromes, which are unable to bind O_2_. The hemichromes interact with the Band3 cytoplasmic tail, also favoring its aggregation and its detachment from the cytoskeleton. The key role of hemoglobin in Band3 clustering is supported by two recent observations: (i) in the early phase of RBC storage, a significant amount of hemoglobin is associated with the lipid bilayer in MVs [127]; (ii) accumulation of oxidized hemoglobin during storage occurs together with its enrichment into MVs [128]. It should be noted that membrane peroxidation also seems to be required for Band3 clustering and termination of the RBC life [129]. Once Band3 is aggregated, it will both (i) promote the binding of autologous immunoglobulin G and initiates the removal of senescent RBCs from the bloodstream [19,130], and (ii) initiate the membrane budding and the subsequent MV release. This vesicle release is most probably due to the membrane:cytoskeleton anchorage destabilization. This involvement of oxidative stresses in vesicle release is supported by the fact that a treatment with antioxidants decreases the formation of MVs from RBCs [131].

•  Calcium Accumulation Model

An alternative model to Band3 aggregation relies on the increase of intracellular Ca^2+^ concentration. This increase is observed during RBC physiological senescence [132], which could partly result from the decreased efficiency of Ca^2+^ extrusion due to the accumulation of oxidative stresses. This increase is also seen in eryptosis (i.e., apoptosis of anucleated cells) which is triggered by a variety of stimuli including hyperosmolarity, oxidative stress and exposure to xenobiotics [133].

To induce Ca^2+^ accumulation inside the RBCs, Ca^2+^ ionophores like A23187 are often used. The sudden increase in Ca^2+^ is known to trigger biochemical and morphological changes that finally result into the release of vesicles. Vesicles collected under this treatment (i) are free of cytoskeleton components, (ii) contain hemoglobin, and (iii) are enriched in GPI-anchored proteins (e.g., acetylcholinesterase and CD55) and raft lipids (Figure 3d). Two types of vesicles differing in size have been described: on one hand MVs with a diameter of ~150 nm and on the other hand nanovesicles (NVs) with a diameter of ~60 nm [67,134,135]. These two types of vesicles can be further distinguished based on biochemical contents. For instance, synexin and sorcin are the most abundant proteins after hemoglobin in NVs, while stomatin is highly enriched in MVs [67]. Two populations of Ca^2+^-induced vesicles differing in size (~200 vs. ~120 nm in diameter) have further been confirmed by another group [136]. The correspondence between the NVs described in [67] with the smallest MV population observed in [136] remains to be determined. Anyway, all these data suggest that different types of vesicles exist [136], which is an additional argument in favor of their formation and shedding from PM specific regions (see Section 5).

Mechanistically, budding and release of MVs under Ca^2+^ increase correlate with the production of diacylglycerol on the inner leaflet and its flop to the outer one [137]. Moreover, altered Ca^2+^ levels induce the recruitment and activation of Ca^2+^-dependent enzymes such as scramblases with subsequent PS externalization, which appears to be one of the main features of MVs, even if PS-negative MVs have been reported [138]. Last but not least, increased Ca^2+^ level activates proteolytic enzymes such as calpains which disrupt the membrane:cytoskeleton connection, favoring vesiculation [67,139] (Figure 4b).

Since Ca^2+^ ionophore-induced MVs and those generated upon senescence and storage differ in protein composition, Bosman et al. suggested that alteration in intracellular Ca^2+^ concentration is not the primary factor in RBC MV generation in vivo nor in the blood banks [21]. We propose the alternative hypothesis that different types of MVs are produced by the same cell, either simultaneously or sequentially, generating MVs with differential lipid and protein compositions. In favor of this hypothesis, Le Van Kim et al. postulated that, only from day 35 of storage, RBCs become very old and exhibit a clustered form of Band3 and membrane microvesiculation [126], which could suggest that another process is responsible for MV release before day 35.

#### 4.1.3. Microvesicles in Red Blood Cell Hemoglobinopathies

The concentration of RBC-derived MVs is increased in the blood of patients with hemoglobinopathies like thalassemia or sickle cell disease [140]. In α- and β-thalassemia, one of the globin chains is mutated leading to an insufficient quantity of hemoglobin heterotetramers and to the formation of hemoglobin precipitates within the erythroid precursors [141]. Blood MVs of those patients contain high concentrations of oxidized denatured globin chains, as well as catalase and peroxiredoxin-2, two enzymes involved in the control of the redox status (Figure 3e). Mechanistically, hemichromes (i.e., oxidized hemoglobin) bind to Band3, inducing the formation of Band3 dimers that are subsequently phosphorylated by tyrosine kinases. According to the Band3 vesiculation model (see Section 4.1.2), this phosphorylation leads to the weakening of the binding between the membrane and the cytoskeleton, as well as the clustering of Band3, finally leading to the membrane instability and the release of MVs [142].

Sickle cell disease is associated with the formation of hemoglobin S (HbS) polymers of deoxygenated hemoglobin. Sickle RBCs were the first pathologic cells described as a source of MVs [140]. Oxidative stress is nowadays recognized as a key component of the chronic inflammatory state associated with sickle cell disease. Reactive oxygen species-mediated damage to sickle RBC membrane proteins and lipids contribute to their rigidity and fragility [143]. It leads to membrane destabilization, poor deformability, changes of the hydration status, increase in intracellular Ca^2+^ and tyrosine phosphorylation of Band3 [110]. One of the consequence is the production of MVs which (i) contain Band3, glycophorin A and protein 4.1 but lack spectrin, (ii) exhibit increased Ca^2+^ level, (iii) contain, but are not enriched in, SM, PC, PS, PE nor in chol, (iv) present a similar acetylcholinesterase activity as in the parental cell membrane, and (vi) contain heme [140,144]. Thus, in haemoglobinopathies, oxidative damage inducing Band3 clustering appears as the initial key step in the microvesiculation process.

#### 4.1.4. Microvesicles in Red Blood Cell Membrane Fragility Diseases

Besides haemoglobinopathies, some membrane fragility diseases like hereditary spherocytosis are associated with an increased vesicle release. This disease is caused by defects in proteins of the ankyrin complexes that vertically connect the membrane to the cytoskeleton [145]. When these interactions are compromised, membrane:cytoskeleton cohesion is lost, leading to membrane destabilization, decrease of the RBC surface area-to-volume ratio with the formation of spherocytes that are trapped and destroyed in the spleen, resulting into hemolysis [146]. Elliptocytosis is another RBC membrane fragility disease that is linked to disruptions of horizontal cytoskeleton interactions, resulting into an alteration of the spectrin tetramer self-association. The RBCs are characterized by an elliptical or elongated shape and by a decreased deformability [147]. Shear-stress induced vesiculation could contribute to membrane loss in this disease but this is not supported by sound evidence.

A recent simulation study nevertheless revealed that (i) vesicles released from spherocytotic and elliptocytotic RBC membranes are more diverse in size than those released from healthy RBCs, and (ii) vesicles released from the elliptocytotic, but not from the spherocytotic, membrane may contain fragments of the cytoskeleton [148] (Figure 3f). However, to the best of our knowledge, no comprehensive analysis of the MV content from the blood of these patients is available in the literature. Even less information is available regarding their biogenesis and shedding from the PM. Several hypotheses have nevertheless been proposed to provide a link between cytoskeleton alteration and vesiculation in spherocytosis. First, since proteins of the ankyrin complex are needed for the vertical anchorage of the membrane to the cytoskeleton, their simple loss could result in reduced mechanical strength and the subsequent vesiculation. Second, secondary loss of cytoskeleton components may create an area of weakness in the membrane. Third, loss of Band3, the most abundant integral membrane protein of the RBC surface, could affect RBC membrane integrity [149]. However, the vesiculation process might differ depending on the underlying molecular defect (i.e., ankyrin, spectrin or Band3 mutation) and thus lead to MVs with different compositions [150]. Accordingly, Band3 has been found in MVs from spherocytotic RBCs with a defect in ankyrin or spectrin, but not from spherocytotic RBCs with a mutation in Band3 [150,151]. A comprehensive study on vesicle composition and shedding mechanisms in spherocytosis and elliptocytosis is therefore required.

### 4.2. Nucleated Cells

Most of the knowledge regarding the mechanism of MV biogenesis in nucleated cells comes from studies on cancerous cells [152,153]. Following cell stimulation, the shedding mechanism seems to start with the influx of Ca^2+^, resulting in the activation of Ca^2+^-dependent proteases, such as calpains. This in turn disrupts the membrane cytoskeleton with formation of membrane protrusions. At the same time, the Ca^2+^-dependent scramblase is activated, leading to PS exposition to the external leaflet [154,155].

To start outward-budding vesiculation at the PM, membrane curvature is required and can be induced by several mechanisms (reviewed in [156]), including changes in lipid composition and asymmetry (see Section 5) and clustering of integral membrane proteins with an inherent curvature. However, little is known about the involvement of these processes in membrane vesiculation and only assumptions can be made. For example, while tetraspanins (integral membrane proteins known to gather together to form PM microdomains [157]) are often proposed as exosomal markers, specialized tetraspanins can also induce PM curvature [158], and their presence in shedding vesicles has been reported [83]. The establishment of a membrane bud could then participate in the sorting of proteins into the shedding MVs. Assisting proteins could also actively help sorting other proteins into MVs. For example, some matrix metalloproteases are delivered to nascent MVs through the association of vesicle-associated membrane protein 3 (VAMP3) with tetraspanin CD9 [159]. Other studies suggest that proteins can be sorted through the endosomal recycling pathway regulated by the GTPase ARF6. This idea arises from the observation that MHC class I, β1-integrin and VAMP3 are contained within MVs and known to be trafficked via ARF6 pathway [16,160].

The mechanisms underlying MV production involve multiple partners, depending on cell type and stimulation. However, Ras superfamily GTPases are postulated to be major mediators of MV formation. Indeed, activated RhoA promotes actin-myosin contraction that is required for MV formation through the downstream signaling of ROCK (Rho-associated coiled-coil containing kinases) and ERK (extracellular signal-regulated kinases) [161]. In cancerous cells in hypoxic conditions, the small GTPase RAB22A colocalizes with budding MVs. Moreover, MV release under hypoxic conditions is completely abrogated upon RAB22A knockdown while it is modestly preserved under non-hypoxic conditions, suggesting that alternative mechanisms exist depending on the hypoxia state of the cell [162]. Muralidharan-Chari et al. showed that ARF6 is responsible for the regulation of MV release in tumor cells. Indeed, once ARF6 is activated, it promotes the recruitment of ERK to the PM. ERK then phosphorylates myosin light-chain kinase which in turn phosphorylates myosin light-chain. This allows the contraction of actomyosin at the necks of MVs and thus MV release [62]. Another pathway implies the endosomal sorting complexes required for transport (ESCRT). This complex was initially thought to only play critical role into exosome biogenesis from the endosomal membrane, but it was later described that some proteins from the ESCRT (named TSG101 and VPS4 ATPase) can be relocated from the endosomal membrane to the PM where they mediate the release of MVs [11]. Accordingly, Booth et al. visualized the budding of domains enriched in proteins from the ESCRT at the lymphocyte PM [9].

## 5. Microvesicle Biogenesis and Shedding—Role of Plasma Membrane Composition and Biophysical Properties

As highlighted above, data available for RBC microvesiculation upon aging in vivo and in vitro and upon Ca^2+^ intracellular boost are rapidly increasing. Accordingly, two models for MV shedding have been proposed (Figure 4a,b). On the other hand, it is not known whether there are different types of EVs that are simultaneously or sequentially released by cells and that could have different roles. Last but not least, the above models do not include the contribution of PM lipids in the vesiculation process. For instance, it is not known whether the budding of MVs could occur from specific regions of the PM and if some specific lipid domains could represent the starting point of the vesiculation process. Although only limited data are available in this respect, most of them appear to converge to the idea that (modulation of) PM lipid content (Section 5.1), transversal asymmetry (Section 5.2) and lateral heterogeneity (Section 5.3) may play a significant role in the vesiculation process.

### 5.1. Plasma Membrane Lipid Composition

As highlighted in Section 3.3, MVs and the PM from which they derive can differ in terms of lipid composition, suggesting a selective sorting into MVs. In this section, we summarize current knowledge of the role of specific SLs and chol in the formation and release of MVs. Comparison to exosome formation is sometimes provided. However, for extended information regarding the role of SLs and chol in the biogenesis of exosomes, please refer to [163,164], respectively.

#### 5.1.1. Sphingolipids and Sphingomyelinases

SLs exhibit both structural and signaling roles. Among SLs, Cer not only serve of structural roles in biomembranes through its conical shape (see below), but also have a variety of effects on signal transduction and the regulation of cell function, particularly the potentiation of signaling pathway leading to cell death. On the other hand, sphingosine 1-phosphate is an important signaling lipid, controlling cell growth, adhesion, migration, survival and inflammatory response, highlighting the importance to maintain an adequate Cer/sphingosine 1-phosphate balance [165]. Among other ways, Cer can be generated upon hydrolysis of SM through the action of SMases. Neutral SMases (n-SMases) are found in the Golgi and the endoplasmic reticulum or in the Golgi and the nucleus but also at the PM [166]. Although n-SMases have been shown to facilitate exosome biogenesis (reviewed in [163]), their role in MV biogenesis has been less explored. One recent study shows that n-SMase inhibition increases the basal release of MVs from epithelial cells while decreasing secretion of exosomes, suggesting that n-SMase differentially controls the release of exosomes and MVs in these cells [167]. This contrasts with the observation by Bianco et al. that n-SMase activity is not required for MV shedding from the cell surface of primary microglia under stimulation with ATP, a very efficient way to promote EV release [168]. Besides n-SMase, acid SMase (a-SMase) is able to generate Cer in lysosomes, but also at other subcellular places. Indeed, an acidic environment also exists outside of lysosomes and the membrane lipid composition could alter the Km of the enzyme, thus allowing a-SMase activity at higher pH [169]. Thus, after certain stimuli, a-SMase can reach the outer PM leaflet by fusion of secretory lysosomes with the PM [170]. Moreover, the enzyme can also be secreted by myeloid cells and the vascular endothelium. The molecular mechanisms behind regulation of a-SMase are only partially characterized (for a review, see [169]). The role of a-SMase in MV shedding has been evidenced in a large diversity of cells under stress membrane conditions (e.g., sickle cell disease, RBCs upon storage) or danger signals (e.g., ATP). For instance, RBC membrane alteration in sickle cell disease enhances SMase activation, resulting in strong increase of production and storage of sphingosine and sphingosine 1-phosphate as well as MV generation. Treatment with amitryptiline, a functional inhibitor of a-SMase, reduces MV generation both in vitro and in vivo. As suggested by the authors, this mechanism could be applicable to other RBC disorders [171]. Acid sphingomyelinase is also implicated in the biogenesis of MVs from RBCs during storage [172] (Figure 4c). Moreover, Bianco et al. have demonstrated that ATP-induced MV shedding by glial cells upon ATP activation of the P_2_X_7_ receptor is associated with the rapid activation and PM translocation of a-SMase [168]. The contribution of a-SMase in MV shedding has been recently confirmed in macrophages upon ATP stimulation [173].

Although the above examples support a role for SMase in MV shedding, the link between Cer production and membrane blebbing is still unclear. Alterations of the PM biophysical properties due to Cer generation could partly provide this link, based on the following features. First, Cer has a cone-shaped structure that can give a spontaneous curvature to the membrane. This property, combined with the fact that Cer synthesized in the external leaflet may be redistributed to the inner one, could lead to membrane evagination [163,174]. Second, hydrolysis of SM, which has a high affinity for chol in membranes, results in increased chol efflux from the PM to intracellular membrane [175] and increased fluidity. Third, several studies report the capacity of Cer to form domains. For instance, real time fluorescence imaging on lipid monolayers treated with SMase has revealed Cer-enriched domains with shape and long-range organization controlled by line tension and dipolar electrostatic repulsion [176]. Hence, hydrophobic Cer molecules separate from other lipids in membranes and self-associate into small Cer-enriched domains which have the tendency to spontaneously fuse into large Cer-enriched platforms easily detectable by fluorescence microscopy (reviewed in [169]). Moreover, although chol- and Cer-enriched domains are dissociated, they are largely interplayed [177] and Cer competes with chol for the formation of domains with SM [178]. Finally, substantial amount of n-SMase PM activity in human skin fibroblasts has been shown to reside at the cytosolic leaflet of SM/Cer-enriched caveolae, suggesting that metabolism of these lipids might occur locally ([166]; see also below).

Besides Cer and sphingosine 1-phosphate, psychosine (also called galactosylsphingosine) has been shown to play a role in MV shedding. This inverted cone-shaped SL progressively accumulates in brain membrane of Krabbe disease (a genetic leukodystrophy due to mutations in the galactosylceramidase gene) and causes demyelination by the killing of oligodendrocytes. Using Twitcher mouse as a model for Krabbe disease, the group of Bongarzone showed ten years ago that psychosine specifically accumulates in lipid rafts. This accumulation occurs together with an increase in chol in these domains, as well as changes in the distribution of the raft markers flotillin-2 and caveolin-1. Altogether, these alterations lead to the deregulation of raft-associated signaling [179]. More recently, the group established a link between raft disruption, membrane microvesiculation and demyelination. For instance, using RBCs and oligodendrocytes, they showed that psychosine disrupts SM-enriched domains and increases the rigidity of local PM areas while promoting the shedding of MVs. Areas of higher rigidity have been confirmed in Twitcher myelin and correlate with higher contents in psychosine and myelin microvesiculation [180] (see also Section 5.3.5).

#### 5.1.2. Cholesterol

As highlighted in Section 3.3, reliable information regarding chol enrichment in MVs as compared to the PM is still lacking. In the same way, only a few studies have been dedicated to the importance of chol to MV biogenesis and shedding, a role that has been proposed for exosome release (reviewed in [164]). Using flow cytometry and EVs stained with anti-chol antibody, Osteikoetxea et al. have shown stronger staining for exosomes than MVs whereas the two populations are enriched in GM1 [87]. Moreover, living keratinocytes labeled by the liquid-disordered (L_d_) marker DiIC18 and the liquid-ordered (L_o_) GM1 marker cholera toxin B subunit reveal submicrometric lipid domain separation together with spontaneous vesiculation of the L_d_ domains and cortical cytoskeleton detachment from the membrane, phenomena enhanced by chol depletion by methyl-β-cyclodextrin (mβCD) [181]. In contrast, depletion of chol from the PM of THP-1 monocytes leads to impaired membrane shedding and reduction of MV abundance [39] while loading of the same cells with chol stimulates MV release [182]. Besides the possibility that high mβCD concentration could affect PM integrity and extract other molecules than chol, one hypothesis to reconcile these observations is the differential chol PM level and PM:cytoskeleton anchorage strength which could strongly vary from one cell to another [183].

Chol could contribute to the microvesiculation process through its ability to modulate PM lipid order and/or via its capacity to cluster into lipid domains. We indeed specifically found that chol-enriched domains are lost by vesiculation upon RBC storage at 4°C, suggesting they could represent sites for vesiculation upon aging. While additional work is needed to demonstrate the contribution of chol in the MV process, our data are supported by theoretical work [184] and biophysical experiments on model membranes [185,186] that have proposed the line tension associated with the domain boundary as driving force for specific lipid domain vesiculation.

### 5.2. Plasma Membrane Transversal Asymmetry

Cell membranes exhibit transbilayer asymmetry, first hypothesized in the 70′s by Bretscher [187]. This asymmetry contributes to PM complexity and diversity by the differential repartition between the two leaflets of lipid molecular shapes, lipid order as well as lipid charge and dipole, thereby leading to optimal physiological output. SM, glycosphingolipids (GSLs) and PC are preferentially found in the outer PM leaflet of mammalian cells whereas PE, PS and phosphatidylinositol-4,5-biphosphate (PIP_2_) are mostly located in the inner leaflet.

Lipid asymmetry participates in several cellular functions involving the formation of high local membrane curvature. For example, inner PIP_2_ contributes to phagocytosis [188]. Moreover, translocation to the outer leaflet of PLPs normally restricted to the inner one (e.g., PE and PS) has been proposed to play a role in a large variety of cell events. First, it participates in hemostasis by regulation of thrombin production [189,190] (see Section 1). Second, appearance of PS and PE at the cell surface often accompanies RBC microvesiculation during storage, resulting into RBC removal [191]. However, as explained at Section 3.3, this is not always the case. Third, PS surface exposure contributes to the clearance of apoptotic cell bodies [192], viewed as a cellular response contributing to the shedding of intracellular Ca^2+^ excess allowing the cell to recover after stress and apoptotic triggers [193]. In parallel to PS externalization, SM flips to the inner leaflet, where it is hydrolyzed to Cer by an intracellular n-SMase. SM hydrolysis disturbs its tight interaction with chol, resulting in chol redistribution from the PM towards the cell interior. Reduced SM and chol contents in turn alter PM biophysical properties, allowing for membrane blebbing and vesicle shedding [194].

### 5.3. Plasma Membrane Lateral Heterogeneity

#### 5.3.1. General Features

Although membrane transversal asymmetry is well accepted, lateral distribution within the same leaflet in lipid domains has been subjected for a long time to intense debates. Limited availability of reliable fluorescent probes, poor lipid fixation, imaging artifacts due to membrane protrusions/projections and utilization of highly disruptive methods of isolation have been often denounced. Moreover, lipid domains have sometimes been reported under non-physiological conditions, potentially explaining the discrepancies between the results obtained by different research groups (reviewed in [183]). Nevertheless, membrane lateral heterogeneity has been shown at different scales, times, compositions and regulations, resulting in a wide diversity of domains in cell membranes. The concept of lipid rafts, introduced in the 90′s by Simons et al. is used to describe unstable (seconds) nanoscale assemblies (<100 nm) enriched in SLs, chol and GPI-anchored proteins [195,196]. Besides rafts, various types of membrane domains are characterized by their enrichment in specific proteins, such as caveolae [197] and tetraspanin-enriched domains [157]. Rafts can sometimes be stabilized to form larger platforms through protein:protein and protein:lipid interactions [198]. In the past decades, owing to the development of new probes and imaging methods, morphological evidence for stable (min vs. sec for rafts) submicrometric domains (>200 nm in diameter vs. <100 nm for rafts) has been reported in artificial models [199,200,201], highly specialized biological membranes [200,202] and a variety of cells from prokaryotes to yeast and mammalian cells [203,204,205,206,207,208,209]. A substantial, albeit non-exhaustive, list of examples is presented in [183] and [210]. Finally, lipid domains could be not stably present but transiently generated by the hydrolysis of specific lipids. An example is the SM degradation by SMase that can form Cer-rich domains with diameters of ~200 nm up to several micrometers [211,212] (see also Section 5.1.1).

It is generally admitted that lipid domains present a different lipid order from the surrounding membrane. This was first suggested in the 90′s by the lipid raft hypothesis, which proposed that sterols and SLs, due to their favorable interactions, can self-aggregate into domains of higher lipid order as compared to the surrounding lipids (bulk membrane). Later, evidence was provided in membrane models. First, sterol-containing biomimetic model membranes, including planar supported lipid layers and giant unilamellar vesicles (GUVs), exhibit the coexistence of two liquid phases, the L_o_ phase enriched in chol and SLs and the L_d_ phase enriched in unsaturated lipids [213,214]. Second, giant PM vesicles (GPMVs) derived from living cells [199] reveal that L_o_ and L_d_ phases in natural PM can assume a wide range of lipid order states [215]. Finally, regions of different lipid order than the bulk have been shown at the surface of several cells including RBCs, platelets and monocytes [216,217,218,219,220] (see below).

Using a combination of mechanical modeling and GUV experiments, Phillips et al. showed that lipid domains can adopt a flat or dimpled morphology, depending on domain spontaneous curvature, boundary line tension and size [221]. Hence, several studies support the possibility that molecule inclusion/exclusion in lipid domains of differential lipid order could provide a way for their sorting into MVs. First, L_d_ phases tend to spontaneously reside in curved membrane regions of GUVs whereas L_o_ phases are preferentially localized in flat regions [185]. Second, modification of the outer PM leaflet by a chol/SM-binding protein (Ostreolysin A) promotes formation of MVs from Madin-Darby canine kidney (MDCK) cells. These MVs exhibit a significant enrichment in lysophosphatidylcholine and chol and could result from the Ostreolysin-binding to chol/SM membrane domains, suggesting that Ostreolysin-induced vesiculation is accompanied by specific lipid sorting into membrane patches that bud from the PM to create vesicles and tubules [222]. Third, submicrometric lipid domain separation together with spontaneous vesiculation of the L_d_ domains has been provided in living keratinocytes labeled by the L_d_ marker DiIC18 and the L_o_ GM1 marker cholera toxin B subunit. Such vesiculation is increased by chol depletion, which further enhances L_o_/L_d_ domain separation and detachment of the cortical cytoskeleton from the membrane [181]. Finally, evidence for lipid domains and their possible role in membrane vesiculation are provided here below for RBCs (Section 5.3.2), platelets (Section 5.3.3), immune cells (Section 5.3.4), nervous cells (Section 5.3.5) and tumor cells (Section 5.3.6). In all these sections, we started by summarizing the state of the art regarding evidence for lipid domains and thereafter provided some clues for their possible involvement in membrane vesiculation.

#### 5.3.2. Red Blood Cells

**Figure 5:**
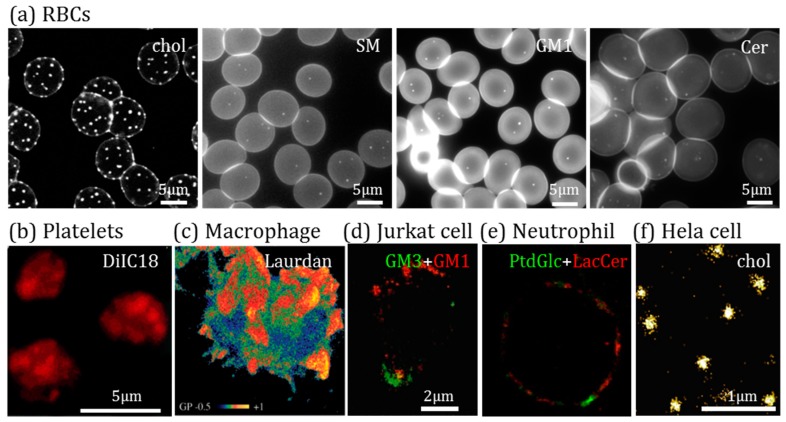
Visualization of plasma membrane lipid domains. (**a**) RBCs labeled for chol with Theta-D4-mCherry or with boron-dipyrromethene-sphingomyelin (BODIPY-SM), -GM1 (BODIPY-GM1) or –ceramide (BODIPY-Cer) and analyzed by fluorescence or confocal microscopy. (**b**) Platelets labeled with DilC18 and analyzed by fluorescence microscopy. (**c**) Macrophage labeled with Laurdan and vizualized by two-photon microscopy. (**d**) Jurkat cell labeled for GM3 and GM1 using anti-GM3 serum and cholera toxin B subunit, respectively, and visualized by confocal imaging. (**e**) Human neutrophil stained for phosphatidylglucoside (PtdGlc) and lactosylceramide (LacCer) and examined by stimulated emission depletion microscopy (STED). (**f**) HeLa cell labeled for chol with Theta-D4-DRONPA and processed by photoactivated localization microscopy (PALM). Adapted from (**a**) [225]; (**b**) [229]; (**c**) [230]; (**d**) [231]; (**e**) [232]; (**f**) [233].

Stable lipid domains at the RBC surface have been evidenced and characterized by our group. They were first revealed by vital fluorescence and/or confocal imaging thanks to the trace insertion at the external PM leaflet of fluorescent analogs of SM, PC, GM1 and Cer (Figure 5a) [204,207,223]. They were later confirmed using fluorescent toxin derivatives specific to endogenous SM [203] and chol (illustrated at Figure 5a for chol) [206], validating a posteriori the use of fluorescent SM analogs to study the behavior of endogenous SM. Those domains are differentially dependent on the chol content, the membrane:cytoskeleton anchorage and the membrane tension. They also differentially associate to high and low RBC membrane curvature areas and exhibit a different response to RBC mechanical stimulation [224]. Altogether, our data suggest the coexistence at the RBC surface of at least three types of domains (i) those mostly enriched in chol, which gather in high-curvature membranes during the RBC deformation, (ii) those mostly enriched in GM1 which might be associated with the Ca^2+^ entry regulation during deformation, and (iii) those mostly enriched in SM and chol, which might regulate the Ca^2+^ efflux during the shape restoration after deformation [183,203,206,210,220,224,225]. Using other probes (e.g., cholera toxin B subunit conjugates, antibodies or treatment with PlcHR2, a phospholipase C/SMase from *Pseudomonas aeruginosa*) and techniques (e.g., SDS-digested freeze-fracture replica labeling), other groups have evidenced stable domains enriched in GM1 [226], GM3 [227] or Cer [228].

Besides those domains, detergent-resistant membranes (DRMs) enriched in stomatin, flotillins, Glut-1, aquaporin-1 and Band3 have been evidenced [234,235]. Those raft-like domains have been linked to the *Plasmodium falciparum* infection [236] and to vesicle release upon Ca^2+^ influx [66,67].

Additional lines of evidence for the existence of lipid domains at the RBC surface are based on multiphoton microscopy of Laurdan-labeled RBCs and AFM imaging of unlabeled RBCs. Domains of various sizes and lipid order have been shown [104,205,220,237,238], suggesting that a large variety of lipid domains might exist at the RBC membrane.

Lutz et al. already provided in 1976 the first indirect clue that RBCs could vesiculate from specific areas of the PM. In fact, they observed that vesicles from sheep RBCs stored at 4°C exhibit the same PLPs as the ghost membrane but a 2-fold increase of lipid-to-protein ratio and an enrichment in glycoproteins, suggesting that those proteins are mobile and can cluster in specific membrane areas, leading to vesiculation [239]. From that time, several observations have supported the potential involvement of lipid domains in RBC membrane vesiculation.

First, DRMs can be prepared from MVs [240] and the MVs present raft-associated lipids and proteins. Indeed, MVs released upon Ca^2+^ increase contain GPI-anchored proteins [241] and stomatin [65,66,67]. Interestingly, RBCs from patients suffering from paroxysmal nocturnal hemoglobinuria do not present GPI-anchored proteins and exhibit a disturbed vesiculation capacity [242]. Moreover, in patients with stomatocytosis associated with stomatin deficiency, Ca^2+^-induced MVs are more numerous and of abnormal size as compared to healthy individuals [243], suggesting that stomatin is important, but not essential, for the regulation of proper MV shedding.

Second, MV shedding is highly dependent on chol, which is essential for both lipid rafts and submicrometric lipid domains. As a matter of fact, Santos et al. have postulated that the total RBC membrane chol content is declined by the release of chol-enriched vesicles [244]. Moreover, upon strong chol depletion (at high mβCD concentrations) in erythroleukemia cells, PS movement to the external leaflet upon addition of a Ca^2+^ ionophore is inhibited [245], suggesting the involvement of lipid domains in transversal lipid asymmetry associated with membrane vesiculation. At low concentrations, mβCD appears to instead increase the number of MVs released [246]. These a priori contradictory observations could partly be explained by the very different mβCD concentration used.

Third, we recently provided clues for the vesiculation of chol- and SM/chol-enriched domains upon RBC storage at 4 °C for <15 days [224]. Our data are, at first glance, in conflict with the observations that (i) RBCs and MVs obtained during storage in blood banks for ~40 days do not exhibit any difference in the main PLP classes (i.e., PC, PE, PS and SM) [92], and (ii) RBCs and MVs from leukoreduced stored RBC units for >50 days show similar PLP composition, except PS [91]. Differences could be related to chol enrichment (not assessed in the two latter studies, to the best of our knowledge), conditions of blood conservation (leukoreduction or not) and/or time of storage (<15 vs. 40 and >50 days). 

Finally, Dinkla et al. showed that the formation of Cer-enriched platforms upon RBC incubation with a-SMase is accompanied by the induction of membrane irregularities enriched in the GPI-anchored protein CD59 [98]. Likewise, upon addition of Cer at the RBC membrane, slow transformation of the biconcave RBC in echinocyte has been shown, suggesting that Cer is responsible for forming membrane spicules thanks to its conical shape [174].

We recently proposed a hypothesis for the control of vesicle formation from lipid domains. By labeling with Laurdan, we revealed that chol-enriched domains exhibit lower lipid order than the rest of the membrane in RBCs at resting state. In contrast, upon RBC vesicle release, the lipid order of the domains increases, leading to a lower lipid order difference between domains and the bulk [220]. We therefore speculated that lipid domains represent specific sites of MV budding by a mechanism driven by the lateral tension applied by the cytoskeleton and its impact on the line tension at phase boundary [220]. A local detachment of the cytoskeleton from the membrane and the echinocytic shape formation seem to be required for normal vesiculation in RBCs. It was already proposed in 2008 that the membrane:cytoskeleton uncoupling favors the coalescence of small rafts into large domains able to curve and to detach from the membrane [240]. Moreover, in comparison to healthy RBCs, fresh spherocytotic RBCs present higher differential lipid order between lipid domains and the bulk membrane together with an accelerated initiation of domain vesiculation upon aging. This suggests that the cytoskeleton pressure could give the main contribution that controls the differential lipid order and drives RBC vesiculation [220].

#### 5.3.3. Platelets

The first proof for lateral lipid heterogeneity at the PM of activated platelets occurred in 1996 when Dorahy et al. isolated at 4°C DRMs enriched in GSLs, chol and GM1 [247]. A few years later, Gousset et al. used fluorescence microscopy and Fourier transform infrared spectroscopy to show that, under platelet activation (either by chilling or thanks to thrombin or collagen), a reversible phase separation in large domains takes place (Figure 5b) [229]. This phase separation, as well as the platelet activation, is highly dependent on the membrane chol content, as both are abrogated by mβCD [248,249]. Those domains have been thought to be involved into platelet early activation by recruiting key receptors like GpVI (a collagen receptor) or FcγRIIa (a low affinity receptor for immune complexes), thus allowing the recruitment and the spatio-temporal activation of tyrosine kinase-dependent pathways [249]. On the other side, chol-enriched domains also appear as sites where the phosphoinositide (PI) metabolism is highly active [250]. The presence of PIP_2_ induces interactions with the actin cytoskeletal elements through their plekstrin homology (PH) domains [251]. Thereby, an artificial enrichment of PIP_2_ in membrane increases the membrane:cytoskeleton energy (and decrease the vesicle release upon activation) while the artificial expression of a PH domain, which sequestrates PIP_2_, results in a reduction of the cytoskeleton membrane adhesion energy [252].

As first evidence for the importance of lipid domains in platelet vesiculation, their activation by thrombin leads to translocation of intracellular stomatin to the PM. Moreover, stomatin is selectively sorted into released MVs while flotillin, another raft marker which exposes different subcellular localization in resting platelets, is excluded [253]. This supports the existence of different types of platelet rafts and maybe vesiculation mechanisms. Several years later, it has been shown that alteration of chol content and/or distribution using mβCD and filipin reduces the release of PS-bearing MVs upon Ca^2+^ activation. While the MVs and the resting platelets present the raft-associated GM1 on their surface, the stimulated platelets from which the MVs originate lose the GM1 signal, suggesting its loss by vesiculation [254].

Lipid rafts in platelets are also connected to platelet membrane deformation. The addition of a low amount of short-chain PLP analogs (1–2% of endogenous PLPs) into the outer leaflet of human resting platelets induces the formation of cell membrane extensions enriched in GM1. Chol depletion impedes formation of these PM extensions, GM1 enrichment and actin polymerization [255]. The same observation has been obtained on thrombin-activated platelets [255]. These data are consistent with the previous observation that chol accumulates in the tips of filopodia and the leading edges of spreading cells [256]. Moreover, PIP kinases are particularly active in lipid domains. After platelet activation and subsequent increase of the intracellular Ca²⁺ concentration and filopodia extension, they are shown to be cleaved by calpain [250]. This would in turn decrease the PIP_2_ levels in domains and *in fine* the adhesion between the membrane and the cytoskeleton at those places, favouring vesicle release [257]. This reinforces the idea that lipid rafts could actively participate to membrane dynamic and actin reorganization.

#### 5.3.4. Immune Cells

Stable lipid domains have been shown on resting macrophages at physiological temperature by two-photon microscopy [230]. Those domains are highly ordered, chol-dependent and grouped at protrusions (filopodia), adhesion points and cell-cell contacts (Figure 5c).

Lipid domains are also relevant to immune cells upon activation. First, in response to chemoattractants, T lymphocytes and neutrophils polarize and migrate, and this is accompanied by lipid coalescence to form domains with different properties at the front and rear of the cell [231,258] (Figure 5d). Although differences have been shown according to cell type and mode of migration (transmigration or migration in a two-dimensional system), domains located at the uropod (rear) are generally (i) resistant to Triton X-100 extraction (and thus highly ordered), (ii) enriched in GM1 and GPI-anchored proteins, and (iii) associated with receptors and signaling molecules involved in cell adhesion [259,260,261]. Regarding domains at the leading edge, they are (i) sensitive to Triton X-100 extraction, as they are not as ordered as uropod domains, (ii) enriched in GM3 and PIP_2_, and (iii) associated with the machinery that induces localized actin polymerization and that senses the environment (G-coupled receptors, PI3kinase, among others) [259,261]. Domains at both cell edges depend on the membrane chol content, and their reversible disappearance inhibits the cell polarization as well as the chemoattractant-stimulated actin polymerization [261].

A second example for the implication of lipid domains in immune cells upon activation occurs in phagocytes once at the site of infection. Nakayama et al. showed that neutrophil phagocytosis signaling through the CD11b/CD18 integrins is dependent on Lyn-coupled lactosylceramide (LacCer)-enriched domains. Those domains colocalize with integrins in actin-enriched phagocytotic cup regions [262]. Moreover, as shown by stimulated emission depletion microscopy (STED), those domains are distinct from phosphatidylglucoside (PtdGlc)-enriched domains, which might on their side mediate the differentiation of neutrophils as well as their apoptosis (Figure 5e) [232].

Chol is not only essential for monocyte deformation [263] but seems also to be involved in their vesiculation, as revealed by the fact that chol depletion decreases MV abundance [39] whereas chol loading stimulates their release [182]. These MVs contain TF and PSGL-1, which are both found in DRMs. Moreover, disruption of rafts by mβCD leads to relocalization of these proteins in non-raft fractions [39].

In ATP-activated macrophages, an elegant study has demonstrated the involvement of lipid rafts in the regulation of membrane receptor trafficking onto filopodia in the course of MV generation. Macrophages release MVs enriched in TF, integrin β1 and PSGL-1 at the tip of filopodia. When filipin is added to perturb raft dynamics, TF translocation onto filopodia and enrichment in MVs are lost, whereas production of PS^+^-MVs and the associated prothrombinase activity are unaltered, suggesting that different mechanisms of sorting and/or MV production also exist in those cells [264]. Another recent study postulates that TF is maintained in a noncoagulant/cryptic state through SM at the PM of macrophages until vesiculation. Accordingly, when macrophages are stimulated with ATP, the a-SMase is translocated to the PM and the hydrolysis of SM increases both TF activity at the cell surface and the release of TF^+^-MVs, without changing the PS externalization. It has been suggested by the authors that inactive TF is associated with rafts and is activated by disruption of these structures [173]. Although at first glance in contradiction with the idea that MVs are shed from lipid domains, this observation could once again be related to the fact that PM exhibits different types of lipid domains with different potential to vesiculate. 

#### 5.3.5. Nervous Cells

DRMs have been evidenced on neurons as well as glial cells. Their composition varies depending on the cell type, the activation state and the pathological conditions. In agreement with observations acquired via the DRM technique, nanometric domains with specific biophysical properties (i.e., stiffer than the surrounding membrane) have been revealed by AFM on hippocampal neurons. Those domains are enriched in GPI-anchored proteins and increase in size without changing in stiffness upon actin depolymerization [265]. In oligodendrocytes, signaling domains enriched in galactosylceramide and sulfatide have also been shown to coalesce in submicrometric domains under activation by apposed membranes in wrapped myelin. This process implies actin filament depolymerization and regulates the lateral diffusion of myelin proteins [266,267]. In microglial cells, the ATP receptor P_2_X_7_ is localized in raft domains [268].

Those lipid domains have been shown to depend on membrane chol, gangliosides and Cer contents [269,270]. Their integrity is essential for major neuronal and glial functions like the neurotransmitter signaling [271], the survival and proliferation signaling in oligodendrocytes [272] and pro-inflammatory response in microglia [270]. As a consequence, they are often associated with neurodegenerative diseases. For example, resulting from a defective SL metabolism, their composition and biophysical properties are altered in the brain cortex from patients suffering from Alzheimer disease. This disruption has been linked to an impaired generation and degradation of amyloid-β peptide [273,274]. In Parkinson’s disease, the mutation of the protein DJ-1, which is associated with rafts in astrocytes, results in raft disruption and consequent glutamatergic signaling disruption [275]. Lipid domains are also affected in lipid-related diseases (e.g., Krabbe disease), where a defective enzymatic activity leads to altered lipid raft signaling pathways [179,276].

In microglial cells, activation of the raft-associated ATP receptor P_2_X_7_ leads to the release of MVs [268] in an a-SMase-dependent manner [168]. In brain tumors, an oncogenic factor is released in MVs containing flotillin-1 [277]. Diseases where the membrane lipid content is altered are also associated with an abnormal vesicle release. For example, in Krabbe disease, the abnormal accumulation of psychosine in the oligodendrocyte membrane disrupts lipid rafts [179]. This leads to the disruption of several signaling pathways but also to an increased rigidity of localized areas promoting the shedding of MVs [180], thought to be essential in the demyelination observed in this disease (see also Section 5.1).

#### 5.3.6. Cancer Cells

Lipid domains have also been observed at the surface of various cancerous cells. First, AFM imaging of purified membranes from human breast cancer cells has revealed the presence of submicrometric domains which contain chol, SM and flotillin 1 [278]. Second, super-resolution fluorescence microscopy of HeLa cells has demonstrated two types of lipid domains of ~250 nm in diameter that are differentially enriched in chol and SM [279] (Figure 5f). Third, electron microscopy of Jurkat T-cells indicates the coexistence of SM- and GM1-enriched domains [280].

Moreover, the lipid domain composition is suggested to be different in the cancerous cells *vs* healthy cells. For example, the carcinoembryogenic antigen (CEA) is not only localized at the apical surface of some colon cancer cells, as in healthy colon epithelial cells, but also at their basolateral side. This observation has been explained by the increased pH within the Golgi which apparently affects GPI-anchorage of CEA in rafts [233]. As another example, Apaf-1 (Apoptotic peptidase activating factor 1, a protein involved in the apoptosome) is abnormally located in rafts instead of in the cytosol in diffuse B cell lymphoma. Upon raft perturbation by mβCD, cytosol location of Apaf-1 is restored and the apoptosome can be correctly assembled [281]. Finally, in multidrug-resistant tumor cells, caveolae-associated caveolin-1, phospholipase D, chol and SM are upregulated, supporting the hypothesis that multidrug-resistance of tumor cells could partially result from lipid domain modifications [282].

As lipid domain modification seems to actively participate to the phenotype of cancerous cells, they could represent interesting targets for anti-cancer treatments. Actually, edelfosine, a synthetic alkyl-lysoPLP with anti-tumor activity, has been shown to destabilize synthetic membranes composed of POPC (palmitoyloleoylphosphatidylcholine)/SM/chol or SM/chol [283]. It preferentially localizes in rafts in lymphoma cells, where it inhibits the PI3K/Akt proliferation signaling pathway and promotes the recruitment of the death receptor Fas [283,284]. Likewise, saponins are widely used in medicine for their anti-cancerous activity [285]. One steroid saponin, ginsenoside Rh2, has been reported to disrupt rafts and to consequently lead to apoptosis, either via the inactivation of the PI3K/Akt pathway [286] or via the activation of the Fas pathway [287]. We recently showed that the activity of ginsenoside Rh2 is decreased by membrane chol, renewing the idea that saponin cytotoxicity is only ascribed to an interaction with membrane chol [288].

Significant evidence supports a role for lipid domains in cancerous MV biogenesis and shedding. For instance, murine leukemia cells release MVs specifically enriched in mammary tumor virus-induced antigens and in chol and SM, suggesting a lipid raft origin [289]. Another example is that the heat shock protein HSP-70-1A, which is located at low concentrations in the cytosol of healthy cells, is upregulated and found at the PM of tumor cells and is associated with increased resistance to radiotherapy and poor survival chances. Biophysical studies on supported lipid bilayers indicate that its membrane location results from interaction with the anionic lipid PS and is enhanced by saturation of the lipid chains as found in dipalmitoylphosphatidylserine (DPPS). Upon addition of chol in the lipid bilayers made of DPPS/DPPC, membrane blebbing occurs [290].

## 6. Future Challenges

As EVs appear to be emerging as targets for disease diagnosis and therapy, a deeper understanding of their composition, biogenesis and shedding mechanisms as well as their pathophysiological roles is required. Whereas EV pathophysiological effects and composition have been extensively studied, biogenesis and shedding mechanisms are still poorly understood. As already stressed by many other reviews, the inherent complications of working with EVs are the extreme versatility of cells to produce different EV subpopulations with distinct sizes, cargoes, morphologies and probably distinct biogenesis mechanisms. This is in addition to the already complicated mixture of EVs in body fluids which contain EVs from different cell origins. In this context, there is a great need to improve the standardization of isolation and analysis of EVs, including identification of specific markers. Hence, once isolated, it is essential to determine MV diversity in a specific physiological process or disease, while providing a comprehensive proteomic and lipidomic analysis in comparison with the PM from which they originate. MVs derived from a same cell type should also be compared in different pathophysiological contexts.

Concerning MV biogenesis, it is still unclear whether a cell is able to simultaneously release distinct populations that contain specific types of molecules or if the content is determined by distinct stimuli, for instance the stage of the disease. Answering these questions will require to determine whether MVs originate from distinct PM areas differentially enriched in specific molecules along physiological processes and disease evolution. The hypothesis behind this is that sorting of proteins or lipids into MVs may rely on their accumulation or exclusion in lipid domains. Several lines of evidence provided in this review support the implication of those domains in membrane vesiculation. First, the lipid raft as MV origin hypothesis is supported by several studies. However, one should remain cautious, since the depletion of membrane chol content to explore the implication of lipid rafts in this process is not sufficient to draw sound conclusions. Second, the biophysical properties of the lipid domains are in accordance with theoretical models and imply the importance of the line tension on domain edge due to differential lipid organization/composition between domains and the bulk membrane. Third, upon membrane:cytoskeleton uncoupling, small rafts are able to coalesce into larger domains able to curve and detach from the membrane, suggesting the interplay between lipid domains and the cytoskeleton in the vesiculation process. Confirming these hypotheses will depend on the development of live cell imaging methods and reliable probes to define the diversity of MVs and to follow the dynamics of lipid domain conversion into MVs.

## Figures and Tables

**Table 1:** Lipid content in microvesicles and enrichment as compared to the originating cells. Data are expressed as percent of total lipid quantified and as MV/cell ratios (brackets), except when specified. Percentages or ratios were calculated from raw data when furnished or estimated on graphs. MS, mass spectrometry; TLC, thin layer chromatography; PM, plasma membrane; -, not determined; SM, sphingomyelin; Cer, ceramide; PC, phosphatidylcholine; PS, phosphatidylserine; PE, phosphatidylethanolamine; chol, cholesterol; PLPs, phospholipids.

Cell Type/Body Fluid	Lipid Analysis	MV Size (nm)	% of Total Lipid Content (MV/Cell Ratio)						Ref.
			SM	Cer	PC	PS	PE	Chol/PLPs	
U87 glioblastoma cells	MS	50–600	15% (2)	0.7% (2.4)	25% (0.9)	17% (1)	9.6% (0.9)	-	[88]
Huh7 hepatocellular carcinoma cells	MS	50–600	15% (3.6)	0.3% (1.5)	27% (1.3)	7.4% (0.6)	6.8% (0.7)	-	[88]
Bone marrow derived stem cells	MS	50–600	8.6% (1.6)	0.5% (1.9)	25% (1.2)	8% (0.7)	3.7% (0.5)	-	[88]
Placenta	MS	-	37%	1%	15%	17%	2%	-	[89]
Plasma EVs (ratio on platelets)	TLC	-	21% (1)	-	59% (1.8)	3.6% (0.3)	9.4% (0.3)	-	[90]
Stored RBCs (53 ± 4 days)	MS	-	(1)	-	(1)	(7)	(0.8)	-	[91]
Stored RBCs (35–42 days)	MS	100–300	33% (1)	-	26% (1)	10% (1)	30% (1)	-	[92]
ATP-depleted RBCs	Enzymatic	~200	26% (0.9)	-	27% (0.9)	-	25% (0.8)	(0.9)	[93]
Platelets	MS	-	14% (1.3)	0.7% (1.3)	27% (0.7)	17% (1)	37% (1)	-	[94]
Platelets (ratio on PM platelets)	TLC	-	25% (1.1)	-	31% (1.1)	14% (0.8)	30% (1)	(1.5)	[95]
